# Attachment and Therapeutic Alliance in Substance Use Disorders: Initial Findings for Treatment in the Therapeutic Community

**DOI:** 10.3389/fpsyt.2021.730876

**Published:** 2021-11-10

**Authors:** Leonie L. Rübig, Jürgen Fuchshuber, Pia Köldorfer, Anita Rinner, Andreas Fink, Human-Friedrich Unterrainer

**Affiliations:** ^1^Institute of Psychology, University of Graz, Graz, Austria; ^2^CIAR: Center for Integrative Addiction Research, Grüner Kreis Society, Vienna, Austria; ^3^Department of Philosophy, University of Vienna, Vienna, Austria; ^4^Department of Psychiatry and Psychotherapeutic Medicine, Medical University Graz, Graz, Austria; ^5^Department of Religious Studies, University of Vienna, Vienna, Austria

**Keywords:** substance use disorder, therapeutic alliance, working alliance dimensions, therapeutic community, attachment

## Abstract

**Background:** There is convincing evidence that individuals suffering from Substance Use Disorder (SUD) often present insecure attachment patterns. In contrast, a strong therapeutic alliance in treatment of SUD has been found to lead to a more positive treatment outcome. However, insecure attachment has been observed to be linked with weaker therapeutic alliance strength. The primary aim of this explorative study was to gain initial insights regarding the influence of attachment and personality characteristics on therapeutic alliance and therapy motivation in SUD patients undergoing treatment at a therapeutic community. Furthermore, SUD patients were compared to healthy controls regarding attachment, personality and mood pathology.

**Methods:** A total sample of 68 participants, 34 inpatients in SUD treatment and 34 age-gender and education adjusted controls, were investigated. Both groups filled in the Adult Attachment Scale (AAS), the Inventory of Personality Organization (IPO-16), and the Brief Symptom Inventory (BSI-18) questionnaires. Additionally, SUD patients filled in the Working Alliance Inventory (WAI-SR) and the adapted German version of the University of Rhode Island Change Assessment scale (FEVER).

**Results:** In line with our assumptions, SUD patients exhibited a decreased amount of attachment security (AAS) which was related to higher personality (IPO-16) and mood pathology (BSI-18). Furthermore, correlational analysis revealed the WAI-SR dimension Bond being positively associated with more secure attachment. A strong task alliance was linked to the Action stage of change (FEVER) and decreased mood but not personality pathology.

**Conclusion:** Our findings confirm the putative negative effect of attachment and personality pathology on therapy motivation and therapeutic alliance in addiction therapy as well as more specifically in therapeutic community treatment. Future research in enhanced samples might focus more on the long-term effects of the interaction of attachment, personality and therapeutic alliance variables.

## Introduction

In 2019, nearly 26,000 people in Austria suffering from Substance Use Disorder (SUD) were in long-term outpatient or inpatient drug-specific treatment. Additionally, there is a highly problematic rate of dropouts around 54% in inpatient drug treatment (1). In contrast, drug treatment retention in terms of the length of stay has been shown to be one of the most important predictors of favorable follow-up treatment outcomes (2, 3). Therefore, keeping patients in treatment is one of the main objectives in SUD therapy and correspondingly, the philosophy of drug-free therapeutic communities is to establish sustainable relationships by building on self-help and mutual-aid between patients. Hereby, the community acts as the central attachment figure, as most of the therapy takes place in group dynamic processes between the clients. Therapists mostly just monitor group dynamics and are mainly responsible for single-therapy sessions (4).

Across various psychotherapeutic approaches, therapeutic alliance is one of the most widely studied process variables in psychotherapy research, mainly because of its obvious link to a positive or negative therapy outcome (5, 6). The concept of therapeutic alliance originates from traditional psychoanalysis and was conceptualized as being closely related to the mechanisms of transference (7). However, early approaches considered therapeutic alliance more as a bonding concept, while Bordin (8) later conceptualized it as a working relationship between client and therapist, emphasizing its cooperative nature (6). Following Bordin's model of alliance as *working alliance)*, it is composed of the three dimensions bond, goals, and tasks, i.e., an affective relationship component, the jointly determined therapy goals, and the process-related tasks of therapist and client (8).

Numerous empirical studies emphasize the substantial relationship between therapeutic alliance and positive treatment outcomes, independent of potential confounders such as cultural background, therapist profile, type of treatment or research design (9–11). This is consistent with the findings from therapy outcome research focusing on SUD treatments (12, 13). Nevertheless, recent studies suggest the relationship between therapeutic alliance and positive outcomes to be lower in SUD patients, by reporting weak correlations of around *r* = 0.14 (9, 14). What is more, various client characteristics have been observed to play an important role for the relationship between therapeutic alliance and the course of SUD treatment. Based on an enhanced literature review, Meier et al. (13, 15) argue that neither SUD patients' demographic nor diagnostic pre-treatment characteristics predicted good therapeutic alliance. However, they found a significant number of studies showing modest relationships for positive previous treatment experience, motivation and treatment readiness, coping strategies, social support and secure attachment.

Attachment pathology has received an increased interest as a potential vulnerability factor in the context of SUD, as insecure attachment patterns can be frequently observed in SUD patients (16–18). In correspondence to this, SUD has been regarded as a certain kind of attachment disorder (19), see also (20) for an enhanced review). Experiences with insufficient attachment figures (in most cases the parents) cause severe emotional disturbances within the child and lead to the formation of deficient internal working models in relation to the self and other people later in life (16, 21–23). Correspondingly, substance use can be described as a kind of “self-medication” in order to regulate affects by means of chemical substances (23, 24). Therefore, from the perspective of attachment theory, therapeutic communities try to break this bond to a harmful substance use or activity and aims to replace it by a bond to the community, with the therapeutic community acting as an attachment figure (19, 25, 26).

Despite of conflicting results [e.g., (26)] attachment organization might have a huge impact regarding an increased alliance, as securely attached individuals were observed to exhibit stronger alliance values than insecurely attached ones (27–30). Correspondingly, Gidhagen et al. (14) reported attachment styles as to be a significant moderator variable between higher working alliance and positive therapy outcome in a sample of SUD patients. Furthermore, there is significant evidence for the substantial connection of attachment organization and personality structure (31). In general, SUDs are often seen as co-occurring with a dysfunctional personality structure as 34–73% of SUD patients were diagnosed for comorbid personality disorder, despite the fact that there is only a prevalence rate of about 10% for the presence of personality disorder in the general population (32–34). What is more, borderline personality organization seems to be associated in particular with the development of SUD (35, 36). In terms of therapy outcome, the occurrence of personality disorders has been shown to be linked to negative treatment outcome like early treatment dropout (12, 37). On the other hand, a strong alliance turned out to be a substantial positive predictor of treatment success (38). However, as personality disorders often cause problematic interpersonal relationships, they can impede the formation of an alliance (39, 40).

Therapy motivation has been reported as a highly important variable as no or low therapy motivation is one of the major challenging problems in the treatment of SUD, throughout the entire therapeutic process. This applies to different phases of therapy as well as to maintaining therapy goals and avoiding relapse. Frequently, treatment is only sought when the physical condition becomes so severely damaged that external help is inevitable, or social pressure becomes too strong. In many cases, inconsistency between actual behavior and a supposedly high verbally communicated therapy motivation can be observed. Once acute problems have subsided, therapy is often terminated prematurely (41).

The transtheoretical model of DiClemente and Prochaska (42) assumes five stages of change in human behavior, each describing the motivational state of the person, as well as the motivation for change (43). As to positive outcomes, low treatment readiness leads to e.g., short-term retention and SUD clients in the pre-contemplation stage are more likely to drop out of therapy prematurely, while clients in the action stage are more likely to actively engage in self-change (43–45). Most importantly, growing evidence has been supporting the idea of readiness for change and certain stages to predict alliance strength (13, 45–47). Ilgen et al. (48) found a positive and strong alliance to be especially important for patients with low therapy motivation, highlighting the potentially beneficial relation between higher stages of change and working alliance. Finally, the putative link regarding SUD and mood pathology has already been extensively investigated in the past and there is evidence that a positive therapeutic alliance might be especially important in keeping SUD clients with additional psychiatric comorbidity in treatment [e.g., (13, 49, 50)].

### Research Aims

Primarily, this study aims to explore the potential link between different attachment dimensions and therapeutic alliance/ therapy motivation in SUD patients undergoing treatment within the surroundings of the therapeutic community. Furthermore, it is intended to compare SUD patients to healthy controls in terms of several parameters of attachment and personality pathology to further examine the assumption of substance misuse as a dysfunctional way of emotion regulation. In line with the primary hypothesis of this study, this investigation might further elucidate the specific challenges regarding the treatment of addiction disorders.

## Method

### Participants and Procedure

A total sample of 68 male (86.8%) and female (13.2%) participants between 20 and 61 years of age (*M* = 33.3, *SD* = 9.7), consisting of one clinical (*n* = 34) and one non-clinical control group (*n* = 34), was investigated. Samples were adjusted in terms of Age, Gender and Education status. All participants of the clinical group were diagnosed for SUD according to the International Classification of Diseases version 10 (ICD 10) (51), by a licensed psychiatrist. These patients were undergoing inpatient therapy in the drug-free environment of an Austrian TC, hosted by the Grüner Kreis society, at the time of the study. In terms of the consumed psychoactive substances the following percentages could be observed: 29.1% Opioids (ICD-Code: F11), 19.0% Alcohol (F10), 19.0% Cannabinoids (F12), 13.9% Sedatives or hypnotics (F13), 11.4% Cocaine (F14) and 7.6% Other stimulants (F15). 76.5% of patients reported poly drug use. Comorbidities with other diagnosis were distributed as follows: 21.4% Affective disorders (F3.x), 16.7% Neurotic, stress-related and somatoform disorders (F4.x), 7.1% Personality and behavioral disorders (F6.x), 7.1% Schizophrenia, schizotypal and delusional disorders (F2.x) and 2.4% Behavioral and emotional disorders with onset usually occurring in childhood and adolescence (F5.x). The sample for the control group was taken from the normal population by means of an internet survey, distributed through social networks. Hereby, the inclusion criterion was an Age range from 18 to 65 years. Exclusion criteria were a diagnosis of SUD and/or any kind of diagnosed mental disorder, either at the time of the study or in the past. Nicotine dependence was not considered in this study. The study protocol was approved by the ethics committee of the University of Graz, Austria. Data of the clinical sample were acquired in a one-time group testing in the therapeutic community in July 2020. Data of the non-clinical sample were collected *via* the online-survey platform LimeSurvey© in November 2020. Written informed consent was given by all participants before answering the questions.

### Psychometric Instruments

#### Mood Pathology

The short version of the *Brief Symptom Inventory* [*BSI-18*; (52); German version: (53)] assesses psychological distress within the past seven days. The inventory includes three subscales: (1) Somatization, (2) Depressiveness and (3) Anxiety. It consists of 18 items in total, which are rated on a 5-point Likert scale ranging from *not at all* (0) to *very much* (4). By summing up all the three scale scores, a Global Severity Index (*GSI*) can be generated that provides information about the overall severity of general psychiatric symptoms. Cronbach's α in this study ranged from 0.75 to 0.84. for the subscales. The total GSI score showed a Cronbach's α of 0.91.

#### Attachment Styles

The German Version of the *Adult Attachment Scale* [*AAS*; (54, 55)] is a self-descriptive measure of attachment-related attitudes consisting of 15 items answered on a 5-point Likert scale, ranging from *strongly disagree* (1) to *strongly agree* (5). The questionnaire is based on Bowlby's attachment theory (56) and consists of three subscales: (1) Anxiety about being rejected or unloved (“Anxiety”), (2) Comfort with closeness and intimacy (“Closeness”) and (3) Comfort in depending on others (“Dependence”). Cronbach's α for the scales ranged from 0.76 to 0.86.

#### Personality Organization

The Inventory of Personality Organization—Short Version [*IPO-16*; (57)] is a self-report instrument to assess personality organization according to Otto Kernberg's model (58). The IPO-16 is composed of 16 items, which are rated on a 5-point Likert scale ranging from *never true* (1) to *always true* (5). The total score is a global measure representing the extent of structural deficit and can serve as an indicator of the presence of a personality disorder. In this study we observed good internal consistency for the scale with a Cronbach's α of 0.88.

#### Readiness to Change

The “*Fragebogen zur Erfassung der Veränderungsbereitschaft”* questionnaire [*FEVER*; (59)] is the German version of the University of Rhode Island Change Assessment Scale [*URICA*; (60)] based on the transtheoretical model of change by DiClemente and Prochaska (42). The FEVER is a self-descriptive procedure to measure readiness to change and to assess therapy motivation in complex problem behaviors. Instead of pointing a person into one single stage, the FEVER provides scores for each of the three temporal-motivational dimensions corresponding to the stages of change: Precontemplation, Contemplation, and Action. The 24 items can be answered on a 5-point Likert scale from *not true at all* (1) to *very true* (5). Cronbach's α for the three scales ranged from 0.80 to 0.84.

#### Therapeutic Alliance

The German client version of the Working Alliance Inventory—short revised [*WAI-SR*; (61)] is based on the frequently used Working Alliance Inventory (*WAI*) by Horvath and Greenberg (62). The WAI is theoretically based on Bordin's (8) conception of the therapeutic alliance and therefore measures the three working alliance dimensions of Bond, Tasks and Goals. The 12 items of the WAI-SR are rated using a 5-point Likert scale ranging from *rarely* (1) to *always* (5). Participants were asked to rate the relationship to their reference therapist. Internal consistency was acceptable to good with Cronbach's α ranging from 0.77 to 0.87.

### Data Analysis

SPSS 26 was used for statistical analyses. For group comparisons, one-way or multivariate analyses of variance and χ^2^ tests were conducted. To investigate the relationship between behavioral measures and the three dimensions of working alliance in the clinical group, Pearson's correlation coefficients were calculated. As the requirement of normal distribution for the use of parametric statistical methods was violated for some variables, equivalent non-parametric procedures were conducted in these cases. Furthermore, in cases where linearity assumption was not given, non-parametric Spearman rank-correlation was also calculated. However, these proceedings did not lead to any deviating result. In order to control for α-inflation, the level of significance was set to *p* < 0.01 in ANOVAs, and Pearson's and Spearman's correlations, while *p*-values < 0.05 were marked as tendencies, but where not further interpreted. Where it was feasible, effect sizes were included.

## Results

### Demographics and Sample Characteristics

Socio-demographic variables of both groups as well as resulting group differences are presented in Table 1.

**Table 1:** Sociodemographic data with group differences.

	**Clinical** **(*****n*** **= 34)**			**Controls (*****n*** **= 34)**			***T***	***df***	***p***
	***M***	***SD***	***Range***	***M***	***SD***	***Range***			
Age	33.9	10.2	(20–61)	32.7	9.2	(20–56)	0.50	66	0.62
Days spent in facility	213.5	158.9	(8–745)	N/A		N/A	
	***n***	***%***			***n***	***%***	**χ** ^2^	***df***	***p***
Gender							0.13	1	0.72
Females	4	11.8			5	14.7			
Males	30	88.2			29	85.3			
Other	0				0				
Education							8.97	4	0.06
No completed education	10	29.4			3	8.8			
Apprenticeship	16	47.1			13	38.2			
Secondary school	2	5.9			7	20.6			
High School	4	11.8			5	14.7			
Bachelor/Master	2	5.9			6	17.6			
Other	0	0			0	0			
Nationality							7.66*	2	0.02
Austria	30	88.2			27	79.4			
Germany	0	0			7	20.6			
Other EU country	1	2.9			0	0			
Non-EU country	3	8.8			0	0			
Occupation							44.54**	5	<
Employed	6	17.6			27	79.4			0.001
Unemployed	22	64.7			1	2.9			
Housewife /- man	0	0			1	2.9			
Retired	5	14.7			0	0			
Student	0	0			5	14.7			
In apprenticeship	1	2.9			0	0			
Family status							18.59**	4	<
Single	21	61.7			8	23.5			0.001
Married	2	5.9			6	17.6			
In partnership	6	17.6			19	55.9			
Divorced	1	2.9			1	2.9			
Seperated	4	11.8			0	0			
Widowed	0	0			0	0			
Parenthood							0.30	1	0.58
No	24	70.6			26	76.5			
Yes	10	29.4			8	23.5			
Therapy phase				N/A		N/A	
Inclusion phase	6	17.6							
Motivation phase	3	8.8							
Aspirant phase	20	58.8							
supervisor phase	5	14.7							

* *p < 0.05*;

** *p < 0.01; N/A, Not Applicable*.

### Group Differences in Behavioral Measures

As shown in Table 2, group comparisons between the clinical and the control group showed that the clinical SUD sample reported significantly higher values on all behavioral dimensions than non-clinical controls did. In correspondence to this, SUD patients exhibited more severe deficits in personality organization, higher mood pathology and less secure attachment attitudes (*F* = 8.99–68.02; η^2^ = 0.12–0.50; all *p* < 0.01).

**Table 2:** Group differences in behavioral measures.

		**Clinical (*****n*** **= 34)**			**Controls** **(*****n*** **= 34)**			**ANOVA**		
	**α**	***M***	***Mdn***	***SD***	***M***	***Mdn***	***SD***	***F (1, 66)***	***η^2^***	***p***
Days spent in facilty		213.5	206.0	158.9			N/A			
Therapy phase			2.0							
**Differences in attachment security: AAS**										
Closeness	0.861	16.5	16.5	4.6	20.7	21.5	3.7	17.66**	0.21	<0.001
Dependence	0.798	16.8	17.0	4.1	20.6	21.0	3.2	18.44**	0.22	<0.001
Anxiety	0.763	11.8	12.0	3.8	8.7	8.0	3.2	13.32*	0.17	0.001
**Differences in psychiatric symptom burden: BSI-18**										
Somatization	0.837	3.4	1.0	4.4	1.1	0.0	1.5	8.99*	0.12	0.01
Depression	0.825	5.5	4.0	4.5	1.8	2.0	1.7	20.58**	0.24	<0.001
Anxiety	0.746	5.5	5.0	3.8	1.8	1.0	1.4	28.15**	0.30	<0.001
GSI	0.907	14.5	12.5	10.7	4.7	4.0	3.4	26.32**	0.29	<0.001
**Differences in personality organization: IPO-16**										
Structural Deficit	0.875	2.4	2.5	0.5	1.6	1.5	0.4	68.02**	0.51	<0.001
**FEVER**										
Precontemplation	0.836	1.9	1.7	0.7			N/A			
Contemplation	0.803	4.3	4.3	0.5		N/A				
Action	0.823	4.0	3.9	0.6						
**WAI-SR**										
Bond	0.847	3.2	3.3	1.0			N/A			
Tasks	0.772	3.3	3.3	0.7		N/A				
Goals	0.869	3.4	3.5	1.0						

* *p < 0.01 ;*

** *p < 0.001; α Cronbach alpha; N/A, Not Applicable; AAS, Adult Attachment Scale; BSI-18, Brief Symptom Inventory; GSI, Global Severity Index; IPO-16, Inventory of Personality Organization; FEVER, Fragebogen zur Erfassung der Veränderungsbereitschaft (URICA); WAI-SR, Working Alliance Inventory—short revised*.

### Correlations Between Behavioral Measures and Working Alliance Dimensions in SUD Patients

As demonstrated in Table 3, we observed the “Bond” dimension of working alliance to be strongly positively associated with the attachment dimension “Dependence” (*r* = 0.61; *p* < 0.001). Furthermore, the “Tasks” dimension was negatively related to BSI subscale “Anxiety” (*r* = −0.44; p < 0.01) and the overall BSI total score “GSI” (*r* = −0.44; *p* < 0.01), as well as “Action” stage of change (*r* = 0.45; *p* < 0.01). No significant relations were found between the “Goals” dimension and any of the examined variables (*p* > 0.01) apart from intercorrelating working alliance dimensions (*p* < 0.01). Also, no significant relations were found between the IPO “Structural deficit” total score and working alliance (*p* > 0.01). The attachment dimension “Dependence” and “Structural deficit” correlated negatively (*r* = −0.47, *p* < 0.01), while “Anxiety” about being rejected or unloved and “Structural deficit” correlated positively (*r* = 0.59; *p* < 0.001). Finally, we observed a positive association between the “Precontemplation” stage and “Structural deficit” (*r* = 0.47; *p* < 0.01). Overall, all significant correlations were moderate to strong. No significant correlations were observed regarding age and sex (all *p* > 0.05).

**Table 3:** Intercorrelations for behavioral measures within the SUD sample (*n* = 34).

	**1**	**2**	**3**	**4**	**5**	**6**	**7**	**8**	**9**	**10**	**11**	**12**	**13**	**14**	**15**	**16**
1. Days spent in facility	–	0.73**	0.08	0.06	−0.12	−0.24	−0.03	−0.14	−0.16	−0.12	−0.26	0.14	0.21	0.16	0.30	0.36^+^
2. Therapy phase		–	0.23	0.24	−0.27	−0.14	−0.29	−0.21	−0.29	−0.18	−0.35	0.21	0.33	0.34	0.41^+^	0.38^+^
AAS																
3. Closeness			–	0.53*	−0.23	−0.09	−0.05	−0.28	−0.15	−0.39^+^	−0.09	−0.21	−0.12	0.41^+^	0.25	0.28
4. Dependence				–	−0.23	−0.36^+^	−0.28	−0.19	−0.33	−0.47*	−0.29	0.11	0.16	0.61**	0.27	0.39^+^
5. Anxiety					–	0.31	0.51*	0.28	0.44*	0.59**	0.47*	−0.08	0.01	−0.30	−0.23	−0.25
BSI-18																
6. Somatization						–	0.41^+^	0.72**	0.84**	0.27	0.26	−0.24	−0.15	−0.17	−0.31	−0.05
7. Depression							–	0.55**	0.79**	0.39^+^	0.24	−0.30	−0.38^+^	−0.33	−0.42^+^	−0.32
8. Anxiety								–	0.89**	0.33	0.02	−0.13	−0.15	−0.08	−0.44*	−0.01
9. GSI									–	0.40^+^	0.21	−0.27	−0.28	−0.24	−0.47*	−0.16
IPO-16																
10. Structural Deficit										–	0.47*	0.11	−0.02	−0.33	−0.20	−0.29
FEVER																
11. Precontemplation											–	−0.46*	−0.47*	−0.33	−0.44^+^	−0.31
12. Contemplation												–	0.81**	0.22	0.38^+^	0.22
13. Action													–	0.33	0.45*	0.34^+^
WAI-SR																
14. Bond														–	0.37^+^	0.72*
15. Tasks															–	0.52*
16. Goals																–

+ * p < 0.05*;

* *p < 0.01*;

** *p < 0.001; AAS, Adult Attachment Scale; BSI-18, Brief Symptom Inventory; GSI, Global Severity Index; IPO-16, Inventory of Personality Organization; FEVER, Fragebogen zur Erfassung der Veränderungsbereitschaft (URICA); WAI-SR, Working Alliance Inventory—short revised*.

## Discussion

In this study it was primarily aimed to explore the putative link between attachment and personality characteristics and task alliance, namely the three dimensions of working alliance “Bond,” “Tasks,” and “Goals,” in SUD patients undergoing treatment within the surroundings of a therapeutic community. In line with our assumptions, attachment security showed a strong positive correlation with the “Bond” dimension. Accordingly, the “Bond” dimension in particular refers to the personal bonding experience between therapist and client. This experience is built on more affective aspects of the therapeutic relationship like confidence, acceptance or mutual trust (7). Moreover, this finding is confirmed by previous research [e.g., (27, 28)].

Furthermore, in order to shed further light on the question why substance use disorders are assumed to be notoriously difficult to treat (1), we investigated possible differences regarding attachment and personality between healthy controls and patients diagnosed with SUD. In correspondence to this, we found SUD patients to differ significantly from an age-gender and education adjusted control group by exhibiting diminished attachment security, as well as higher amounts of personality and mood pathology. These findings are highly consistent with the literature [e.g., (16, 17, 34, 35, 49, 63)].

In addition, resonating with previous studies, we found an increased personality structural deficit to be related to more insecure attachment [e.g., (31)]. More in general, these findings further underline the well-established assumption of substance misuse as a dysfunctional way of emotion regulation (63, 64). Taken together, these results seem to suggest that one of the difficulties in the treatment of SUD might be related to addiction specific problems regarding attachment security. However, further research needs to be done, comparing different patient populations (e.g. SUD patients vs. mood disorder patients) to further investigate this assumption.

Moreover, we explored correlation patterns of attachment and personality dysfunctioning, mood pathology, and therapy motivation readiness in SUD patients. As to mood pathology, clients who felt less anxious and demonstrated a less severe overall mood pathology rated the “Tasks” working alliance with their therapist to be moderately stronger. We only found clients in the “Action” stage of treatment readiness to exhibit a higher “Tasks” working alliance with their therapists. This relates to findings of Fitzpatrick and Irannejad (65) who found higher stages of change in adolescents being strongly related to higher “Tasks” and “Goals” working alliance, highlighting their collaborative nature. More in general, our results support the idea of a potentially beneficial relation between higher stage of change and working alliance. Interestingly, mood disorder related symptoms like anxiety often affect motivation and clients in a higher stage of change have been observed to exhibit stronger working alliance and symptom improvement (46, 47). In contrast, we observed that clients in “Precontemplation” stage exhibited a more severely impaired personality structure and described themselves as feeling more anxious about being rejected or unloved. This aspect of insecure attachment was also related to an increased mood pathology. The findings suggest that less pathology in patients is associated with higher treatment readiness. Consequently, they could have been more capable to engage in a strong working alliance. On the other hand, a weak working alliance would likely not have contributed to symptom improvement. Therefore, our results might reflect these connections. Against our expectations, personality pathology was not significantly correlated to working alliance dimensions. This finding is in contrast to previous results, where higher levels of impaired personality organization impeded working alliance formations in residential treatment of SUD (40).

Our study offers additional insights into the putative effect of attachment and personality pathology on working alliance and therapy motivation in SUD patients, by expanding these findings for the therapeutic community environment. In this study we focused on the examination of alliances between SUD clients, but further research is needed to investigate within-patient therapeutic alliance. This might contribute to more knowledge regarding the specific direction of the association between therapeutic alliance and symptomatic improvement, as previously pointed out by Gidhagen et al. (14). Does a stronger therapeutic alliance lead to symptomatic improvement or does the decrease of symptoms increase therapeutic alliance? Current studies mostly find therapeutic alliance to predict symptom improvement, but an interplay is also very likely, especially regarding common therapeutic alliance fluctuation and ruptures throughout treatment (7, 11, 66).

### Limitations and Future Perspectives

Due to the exploratory nature of this study, its main limitation is the small number of participants. Moreover, since inconsistent therapeutic alliance developments have been observed, a longitudinal study approach applying therapeutic alliance oriented measures might probably deliver in-depth insights into its development over time, providing a trait-like characteristic instead of state-alliance (5, 7). Additionally, only self-report measures were used in this study. As to therapeutic alliance measures, previous research found client-rated therapeutic alliance to be most predictive for outcome variables like dropout [e.g., (67)]. However, in fact therapists' assessment of therapeutic alliance has sometimes been found to be a more precise predictor [e.g., (68)]. Therefore, it would be appealing for future research to investigate possible deviations in client and therapist perception of their therapeutic alliance. Likewise, this study did not investigate the impact of same-gender or different-gender therapist/patient pairings, which might have had an influence on therapeutic alliance. Hence, further studies should consider the patient-therapist gender match as a potential confounding variable. Another limitation of this study is that neither severity nor duration of the SUD diagnoses were assessed in our patient sample. It is also important to state that individuals suffering from SUD form a quite heterogenous group often presenting a variety of comorbid psychiatric disorders (50). This circumstance might also have affected our findings. A study with larger samples would make it possible to differentiate between specific SUD diagnoses. Similarly, due to the small sample size of controls diagnosed with mood disorders, we were unable to include these participants as a separate group. Hence, to avoid a possible confounding variable in our control group we decided to exclude these participants. Nevertheless, comparisons of participants with mood disorders and patients suffering from SUD might be a particularly interesting research topic, as mood disorders pose a significant vulnerability to develop addictions (64).

Finally, literature suggests the associations between therapeutic alliance and attachment to be more complex with various intervariable connections [e.g., (14, 27)]. In order to gain an enhanced understanding of the relationship between working alliance dimensions and attachment, as well as personality, mood pathology and therapy motivation, future research might focus on more complex research designs in SUD samples. Also, it would be feasible to include therapy outcome as a dependent variable and to examine possible mediational relationships regarding attachment, personality organization and therapeutic alliance. In correspondence to this, other variables that have been observed to play a role in the attachment-alliance linkage—such as the type of experienced abuse—should be considered in future studies [e.g., (14)].

## Conclusion

Still, some notable implications for treatment of SUD can be derived from our findings: They highlight once more the importance of therapeutic alliance as a beneficial relationship between client and therapist in SUD treatment. However, regarding the diminished attachment security often found in SUD patients, this might be an especially challenging task for practicians working with this patient population. In conclusion, our findings point toward the need to take the client's attachment style into account while establishing the therapeutic alliance and to carefully consider related pathologies and motivational aspects.

## Data Availability Statement

The raw data supporting the conclusions of this article will be made available by the authors, without undue reservation.

## Ethics Statement

The studies involving human participants were reviewed and approved by University of Graz. The patients/participants provided their written informed consent to participate in this study.

## Author Contributions

LR, AF, and H-FU conceptualized the study. LR, AR, and PK collected the data. LR and JF analyzed and interpreted the data. LR and H-FU drafted the manuscript. JF, AR, PK, AF, and H-FU critically reviewed it. All authors gave their final approval of the manuscript.

## Conflict of Interest

The authors declare that the research was conducted in the absence of any commercial or financial relationships that could be construed as a potential conflict of interest.

## Publisher's Note

All claims expressed in this article are solely those of the authors and do not necessarily represent those of their affiliated organizations, or those of the publisher, the editors and the reviewers. Any product that may be evaluated in this article, or claim that may be made by its manufacturer, is not guaranteed or endorsed by the publisher.
